# (*R*)-2-Methyl­piperazine-1,4-diium diaqua­tetra­chloridoferrate(II)

**DOI:** 10.1107/S1600536810035506

**Published:** 2010-09-08

**Authors:** Cong-Hu Peng, Yun-Peng Li

**Affiliations:** a Department of Chemical and Environmental Engineering, Anyang Institute of Technology, Anyang 455000, People’s Republic of China; b Anyang Administration of Work Safety, Henan Province 455000, People’s Republic of China

## Abstract

In the title salt, (C_5_H_14_N_2_)[FeCl_4_(H_2_O)_2_], the Fe^II^ cation is coordinated by four Cl^−^ anions and two water mol­ecules in a distorted octa­hedral geometry. The piperazine ring adopts a normal chair conformation. Inter­molecular N—H⋯Cl, N—H⋯(Cl,Cl) and O—H⋯Cl hydrogen bonding is present in the crystal structure.

## Related literature

For hydrogen bonding in metal–chlorido complexes, see: Brammer *et al.* (2001 ▶); Bremner & Harrison (2003 ▶); Kefi & Nasr (2005 ▶). For the crystal structure of a related compound, piperazindiium tetra­chloridozincate(II), see: Sutherland & Harrison (2009 ▶).
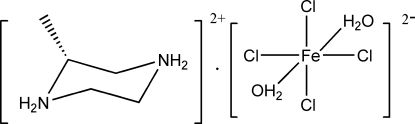


## Experimental

### 

#### Crystal data

(C_5_H_14_N_2_)[FeCl_4_(H_2_O)_2_]
*M* *_r_* = 335.86
Monoclinic, 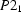

*a* = 8.6013 (17) Å
*b* = 6.4495 (13) Å
*c* = 12.024 (2) Å
β = 101.64 (3)°
*V* = 653.3 (2) Å^3^
*Z* = 2
Mo *K*α radiation
μ = 1.95 mm^−1^
*T* = 291 K
0.28 × 0.24 × 0.20 mm

#### Data collection

Rigaku SCXmini diffractometer
Absorption correction: multi-scan (*CrystalClear*; Rigaku, 2005 ▶) *T*_min_ = 0.8, *T*_max_ = 0.9
6105 measured reflections
2558 independent reflections
2456 reflections with *I* > 2σ(*I*)
*R*_int_ = 0.025

#### Refinement

*R*[*F* ^2^ > 2σ(*F* ^2^)] = 0.023
*wR*(*F* ^2^) = 0.050
*S* = 1.08
2558 reflections
129 parameters
1 restraint
H-atom parameters constrained
Δρ_max_ = 0.19 e Å^−3^
Δρ_min_ = −0.24 e Å^−3^
Absolute structure: Flack (1983 ▶), 1156 Friedel pairs
Flack parameter: 0.010 (14)

### 

Data collection: *CrystalClear* (Rigaku, 2005 ▶); cell refinement: *CrystalClear*; data reduction: *CrystalClear*; program(s) used to solve structure: *SHELXS97* (Sheldrick, 2008 ▶); program(s) used to refine structure: *SHELXL97* (Sheldrick, 2008 ▶); molecular graphics: *SHELXTL* (Sheldrick, 2008 ▶); software used to prepare material for publication: *SHELXL97*.

## Supplementary Material

Crystal structure: contains datablocks I, global. DOI: 10.1107/S1600536810035506/xu5019sup1.cif

Structure factors: contains datablocks I. DOI: 10.1107/S1600536810035506/xu5019Isup2.hkl

Additional supplementary materials:  crystallographic information; 3D view; checkCIF report

## Figures and Tables

**Table 1:** Hydrogen-bond geometry (Å, °)

*D*—H⋯*A*	*D*—H	H⋯*A*	*D*⋯*A*	*D*—H⋯*A*
N1—H1*C*⋯Cl2^i^	0.90	2.62	3.443 (2)	152
N1—H1*C*⋯Cl4^i^	0.90	2.81	3.379 (3)	122
N1—H1*D*⋯Cl4^ii^	0.90	2.28	3.169 (3)	167
N2—H2*C*⋯Cl1^iii^	0.90	2.26	3.145 (3)	168
N2—H2*D*⋯Cl3	0.90	2.45	3.275 (2)	152
O1—H11⋯Cl3^iv^	0.82	2.33	3.147 (2)	173
O1—H12⋯Cl3^v^	0.89	2.24	3.127 (2)	176
O2—H21⋯Cl2^iii^	0.93	2.19	3.119 (2)	174
O2—H22⋯Cl2^vi^	0.86	2.31	3.1590 (18)	168

Symmetry codes: (i) 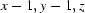; (ii) 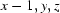; (iii) 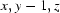; (iv) 
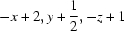
; (v) 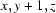; (vi) 
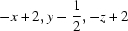
.

**Fig. 1.**
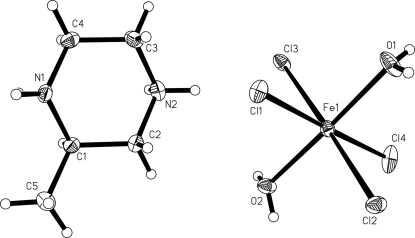
The asymmetric unit of the title compound with atom labels. Displacement ellipsoids were drawn at the 30% probability level

**Fig. 2.**
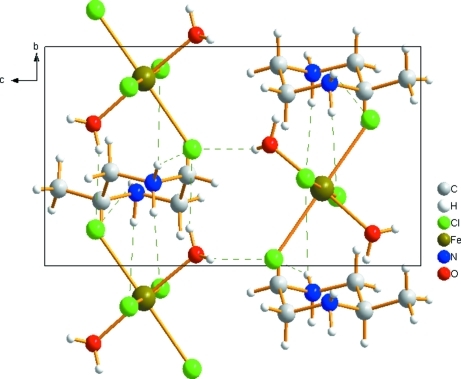
The packing viewed along the a axis. Hydrogen bonds are drawn as dashed lines

## supplementary crystallographic information

### Comment

Recently much attention has been devoted to hydrogen bonding networks in molecular salts containing metal-chlorido complexes (Brammer *et al.*, 2001; Bremner & Harrison, 2003; Kefi & Nasr, 2005). The crystal structure of piperazinediium tetrachloridozincate(II) has been reported (Sutherland & Harrison, 2009). The construction of new members of this family is an important direction in the development of coordination chemistry. We report here the crystal structure of the title compound.

The crystal structure of the title compound (Fig. 1) contains the protonated piperazindiium cations and trans-Fe(H_2_O)_2_Cl_4_ octahedral anions. The piperazine ring adopts a chair conformation. An extensive network of N—H···Cl, N—H··· (Cl,Cl) and O—H···Cl hydrogen bonds results in a structure with a three-dimensional hydrogen-bond network (Fig. 2).

### Experimental

(*R*)-2-Methylpiperazine (2 mmol, 0.2 g), FeCl_3_(2 mmol, 0.31 g), KI (1 mmol, 0.17), I_2_ (0.5 mmol, 0.13 g) and 5% aqueous HCl (5 ml) were dissolved in 10 ml water, the solution was heated to 353 K (0.5 h), forming a clear solution. The reaction mixture was cooled slowly to room temperature, crystals of the title compound were formed after 6 d.

### Refinement

Water H atoms were located in a difference Fourier map and refined as riding their as found relative positions with U_iso_(H) = 1.5U_eq_(O). Other H atoms were placed in calculated positions with C—H = 0.9 or 0.98 and N—H = 0.90 Å, and refined using a riding model, with U_iso_(H) = 1.2U_eq_(C,N).

### Crystal data

**Table:**

(C_5_H_14_N_2_)[FeCl_4_(H_2_O)_2_]	*F*(000) = 344
*M**_r_* = 335.86	*D*_x_ = 1.707 Mg m^−^^3^
Monoclinic, *P*2_1_	Mo *K*α radiation, λ = 0.71073 Å
Hall symbol: P 2yb	Cell parameters from 2456 reflections
*a* = 8.6013 (17) Å	θ = 3.2–26.0°
*b* = 6.4495 (13) Å	µ = 1.95 mm^−^^1^
*c* = 12.024 (2) Å	*T* = 291 K
β = 101.64 (3)°	Block, yellow
*V* = 653.3 (2) Å^3^	0.28 × 0.24 × 0.20 mm
*Z* = 2	

### Data collection

**Table:**

Rigaku SCXmini diffractometer	2558 independent reflections
Radiation source: fine-focus sealed tube	2456 reflections with *I* > 2σ(*I*)
graphite	*R*_int_ = 0.025
Detector resolution: 13.6612 pixels mm^-1^	θ_max_ = 26.0°, θ_min_ = 3.2°
ω scans	*h* = −10→10
Absorption correction: multi-scan (*CrystalClear*; Rigaku, 2005)	*k* = −7→7
*T*_min_ = 0.8, *T*_max_ = 0.9	*l* = −14→14
6105 measured reflections	

### Refinement

**Table:**

Refinement on *F*^2^	Hydrogen site location: inferred from neighbouring sites
Least-squares matrix: full	H-atom parameters constrained
*R*[*F*^2^ > 2σ(*F*^2^)] = 0.023	*w* = 1/[σ^2^(*F*_o_^2^) + (0.0172*P*)^2^ + 0.0801*P*] where *P* = (*F*_o_^2^ + 2*F*_c_^2^)/3
*wR*(*F*^2^) = 0.050	(Δ/σ)_max_ = 0.001
*S* = 1.08	Δρ_max_ = 0.19 e Å^−^^3^
2558 reflections	Δρ_min_ = −0.24 e Å^−^^3^
129 parameters	Extinction correction: *SHELXL97* (Sheldrick, 2008), Fc^*^=kFc[1+0.001xFc^2^λ^3^/sin(2θ)]^-1/4^
1 restraint	Extinction coefficient: 0.116 (2)
Primary atom site location: structure-invariant direct methods	Absolute structure: Flack (1983), 1156 Friedel pairs
Secondary atom site location: difference Fourier map	Flack parameter: 0.010 (14)

### Special details

**Table:**

Geometry. All esds (except the esd in the dihedral angle between two l.s. planes) are estimated using the full covariance matrix. The cell esds are taken into account individually in the estimation of esds in distances, angles and torsion angles; correlations between esds in cell parameters are only used when they are defined by crystal symmetry. An approximate (isotropic) treatment of cell esds is used for estimating esds involving l.s. planes.
Refinement. Refinement of *F*^2^ against ALL reflections. The weighted *R*-factor *wR* and goodness of fit *S* are based on *F*^2^, conventional *R*-factors *R* are based on *F*, with *F* set to zero for negative *F*^2^. The threshold expression of *F*^2^ > σ(*F*^2^) is used only for calculating *R*-factors(gt) *etc*. and is not relevant to the choice of reflections for refinement. *R*-factors based on *F*^2^ are statistically about twice as large as those based on *F*, and *R*- factors based on ALL data will be even larger.

### Fractional atomic coordinates and isotropic or equivalent isotropic displacement parameters (Å^2^)

**Table:**

	*x*	*y*	*z*	*U*_iso_*/*U*_eq_	
Fe1	0.92584 (4)	0.85265 (6)	0.73702 (3)	0.02261 (10)	
Cl1	0.63527 (7)	0.89798 (9)	0.69296 (5)	0.03230 (17)	
Cl2	0.95176 (8)	1.16607 (10)	0.86527 (5)	0.03236 (16)	
Cl3	0.88976 (8)	0.53109 (10)	0.60858 (5)	0.03094 (16)	
Cl4	1.21704 (8)	0.81475 (10)	0.77621 (7)	0.0463 (2)	
N1	0.2717 (2)	0.3313 (4)	0.75683 (15)	0.0271 (5)	
H1C	0.1922	0.2493	0.7690	0.033*	
H1D	0.2396	0.4639	0.7587	0.033*	
N2	0.5845 (2)	0.3786 (4)	0.71209 (16)	0.0309 (5)	
H2C	0.6147	0.2453	0.7103	0.037*	
H2D	0.6648	0.4588	0.6994	0.037*	
O1	0.9382 (2)	1.0515 (3)	0.59861 (15)	0.0433 (5)	
H11	0.9818	1.0347	0.5446	0.065*	
H12	0.9188	1.1870	0.6013	0.065*	
O2	0.91049 (19)	0.6462 (3)	0.87073 (13)	0.0303 (4)	
H21	0.9234	0.5037	0.8634	0.045*	
H22	0.9545	0.6697	0.9406	0.045*	
C1	0.4148 (3)	0.2974 (4)	0.84969 (18)	0.0242 (5)	
H1A	0.4447	0.1506	0.8508	0.029*	
C2	0.5510 (3)	0.4269 (4)	0.82553 (19)	0.0271 (6)	
H2A	0.5248	0.5727	0.8289	0.032*	
H2B	0.6452	0.4003	0.8833	0.032*	
C3	0.4415 (3)	0.4152 (5)	0.6213 (2)	0.0339 (6)	
H3A	0.4649	0.3796	0.5480	0.041*	
H3B	0.4127	0.5607	0.6198	0.041*	
C4	0.3052 (3)	0.2856 (4)	0.64264 (19)	0.0317 (6)	
H4A	0.2115	0.3146	0.5849	0.038*	
H4B	0.3308	0.1398	0.6379	0.038*	
C5	0.3760 (3)	0.3539 (5)	0.9633 (2)	0.0399 (6)	
H5A	0.3347	0.4926	0.9600	0.060*	
H5B	0.4705	0.3458	1.0213	0.060*	
H5C	0.2981	0.2593	0.9807	0.060*	

### Atomic displacement parameters (Å^2^)

**Table:**

	*U*^11^	*U*^22^	*U*^33^	*U*^12^	*U*^13^	*U*^23^
Fe1	0.02514 (17)	0.01809 (17)	0.02560 (17)	−0.00031 (12)	0.00748 (12)	0.00170 (13)
Cl1	0.0251 (3)	0.0260 (4)	0.0452 (4)	0.0010 (2)	0.0056 (2)	−0.0016 (3)
Cl2	0.0461 (4)	0.0235 (3)	0.0255 (3)	0.0002 (3)	0.0024 (3)	−0.0033 (3)
Cl3	0.0468 (4)	0.0231 (3)	0.0269 (3)	0.0018 (3)	0.0171 (3)	−0.0002 (3)
Cl4	0.0270 (4)	0.0260 (5)	0.0866 (6)	0.0004 (3)	0.0131 (3)	0.0033 (4)
N1	0.0224 (10)	0.0257 (12)	0.0326 (11)	−0.0024 (9)	0.0039 (8)	0.0032 (10)
N2	0.0295 (11)	0.0285 (13)	0.0388 (11)	−0.0037 (10)	0.0163 (8)	−0.0018 (11)
O1	0.0727 (14)	0.0263 (10)	0.0418 (11)	0.0091 (10)	0.0376 (10)	0.0080 (9)
O2	0.0425 (10)	0.0245 (10)	0.0224 (8)	−0.0018 (8)	0.0033 (7)	0.0017 (8)
C1	0.0243 (12)	0.0214 (14)	0.0249 (12)	−0.0003 (10)	0.0005 (9)	0.0041 (10)
C2	0.0235 (12)	0.0272 (15)	0.0300 (13)	−0.0037 (11)	0.0044 (10)	−0.0014 (11)
C3	0.0438 (15)	0.0347 (16)	0.0247 (12)	−0.0036 (12)	0.0106 (10)	0.0005 (11)
C4	0.0350 (14)	0.0298 (15)	0.0273 (13)	−0.0028 (11)	−0.0007 (10)	−0.0031 (11)
C5	0.0432 (15)	0.0486 (17)	0.0298 (13)	−0.0074 (14)	0.0122 (11)	0.0019 (13)

### Geometric parameters (Å, °)

**Table:**

Fe1—O2	2.1122 (17)	O2—H21	0.9315
Fe1—O1	2.1205 (18)	O2—H22	0.8622
Fe1—Cl1	2.4654 (9)	C1—C2	1.514 (3)
Fe1—Cl4	2.4655 (8)	C1—C5	1.515 (3)
Fe1—Cl2	2.5249 (9)	C1—H1A	0.9800
Fe1—Cl3	2.5669 (8)	C2—H2A	0.9700
N1—C4	1.488 (3)	C2—H2B	0.9700
N1—C1	1.502 (3)	C3—C4	1.503 (3)
N1—H1C	0.9000	C3—H3A	0.9700
N1—H1D	0.9000	C3—H3B	0.9700
N2—C2	1.483 (3)	C4—H4A	0.9700
N2—C3	1.490 (3)	C4—H4B	0.9700
N2—H2C	0.9000	C5—H5A	0.9600
N2—H2D	0.9000	C5—H5B	0.9600
O1—H11	0.8195	C5—H5C	0.9600
O1—H12	0.8919		
			
O2—Fe1—O1	177.97 (9)	H21—O2—H22	103.2
O2—Fe1—Cl1	91.26 (5)	N1—C1—C2	109.07 (19)
O1—Fe1—Cl1	88.22 (6)	N1—C1—C5	109.81 (19)
O2—Fe1—Cl4	90.52 (5)	C2—C1—C5	111.1 (2)
O1—Fe1—Cl4	90.00 (6)	N1—C1—H1A	108.9
Cl1—Fe1—Cl4	178.22 (3)	C2—C1—H1A	108.9
O2—Fe1—Cl2	92.94 (5)	C5—C1—H1A	108.9
O1—Fe1—Cl2	89.03 (6)	N2—C2—C1	111.2 (2)
Cl1—Fe1—Cl2	89.83 (3)	N2—C2—H2A	109.4
Cl4—Fe1—Cl2	90.09 (3)	C1—C2—H2A	109.4
O2—Fe1—Cl3	85.99 (5)	N2—C2—H2B	109.4
O1—Fe1—Cl3	92.03 (6)	C1—C2—H2B	109.4
Cl1—Fe1—Cl3	88.41 (3)	H2A—C2—H2B	108.0
Cl4—Fe1—Cl3	91.70 (3)	N2—C3—C4	110.1 (2)
Cl2—Fe1—Cl3	177.92 (3)	N2—C3—H3A	109.6
C4—N1—C1	112.03 (19)	C4—C3—H3A	109.6
C4—N1—H1C	109.2	N2—C3—H3B	109.6
C1—N1—H1C	109.2	C4—C3—H3B	109.6
C4—N1—H1D	109.2	H3A—C3—H3B	108.2
C1—N1—H1D	109.2	N1—C4—C3	110.5 (2)
H1C—N1—H1D	107.9	N1—C4—H4A	109.5
C2—N2—C3	110.8 (2)	C3—C4—H4A	109.5
C2—N2—H2C	109.5	N1—C4—H4B	109.5
C3—N2—H2C	109.5	C3—C4—H4B	109.5
C2—N2—H2D	109.5	H4A—C4—H4B	108.1
C3—N2—H2D	109.5	C1—C5—H5A	109.5
H2C—N2—H2D	108.1	C1—C5—H5B	109.5
Fe1—O1—H11	130.2	H5A—C5—H5B	109.5
Fe1—O1—H12	121.6	C1—C5—H5C	109.5
H11—O1—H12	106.1	H5A—C5—H5C	109.5
Fe1—O2—H21	121.5	H5B—C5—H5C	109.5
Fe1—O2—H22	123.2		
			
C4—N1—C1—C2	55.9 (3)	C5—C1—C2—N2	−177.4 (2)
C4—N1—C1—C5	177.8 (2)	C2—N2—C3—C4	−57.9 (3)
C3—N2—C2—C1	58.3 (3)	C1—N1—C4—C3	−56.9 (3)
N1—C1—C2—N2	−56.3 (3)	N2—C3—C4—N1	56.9 (3)

### Hydrogen-bond geometry (Å, °)

**Table:**

*D*—H···*A*	*D*—H	H···*A*	*D*···*A*	*D*—H···*A*
N1—H1C···Cl2^i^	0.90	2.62	3.443 (2)	152
N1—H1C···Cl4^i^	0.90	2.81	3.379 (3)	122
N1—H1D···Cl4^ii^	0.90	2.28	3.169 (3)	167
N2—H2C···Cl1^iii^	0.90	2.26	3.145 (3)	168
N2—H2D···Cl3	0.90	2.45	3.275 (2)	152
O1—H11···Cl3^iv^	0.82	2.33	3.147 (2)	173
O1—H12···Cl3^v^	0.89	2.24	3.127 (2)	176
O2—H21···Cl2^iii^	0.93	2.19	3.119 (2)	174
O2—H22···Cl2^vi^	0.86	2.31	3.1590 (18)	168

Symmetry codes: (i) *x*−1, *y*−1, *z*; (ii) *x*−1, *y*, *z*; (iii) *x*, *y*−1, *z*; (iv) −*x*+2, *y*+1/2, −*z*+1; (v) *x*, *y*+1, *z*; (vi) −*x*+2, *y*−1/2, −*z*+2.
